# Potential T Cell-Intrinsic Regulatory Roles for IRF5 via Cytokine Modulation in T Helper Subset Differentiation and Function

**DOI:** 10.3389/fimmu.2020.01143

**Published:** 2020-06-03

**Authors:** Zarina Brune, Matthew R. Rice, Betsy J. Barnes

**Affiliations:** ^1^Zucker School of Medicine at Hofstra/Northwell, Hempstead, NY, United States; ^2^Center for Autoimmune Musculoskeletal and Hematopoietic Diseases, The Feinstein Institutes for Medical Research, Manhasset, NY, United States; ^3^Departments of Molecular Medicine and Pediatrics, Zucker School of Medicine at Hofstra/Northwell, Hempstead, NY, United States

**Keywords:** autoimmune disease, Th1, Th2, Th17, polarization

## Abstract

Interferon Regulatory Factor 5 (IRF5) is one of nine members of the IRF family of transcription factors. Although initially discovered as a key regulator of the type I interferon and pro-inflammatory cytokine arm of the innate immune response, IRF5 has now been found to also mediate pathways involved in cell growth and differentiation, apoptosis, metabolic homeostasis and tumor suppression. Hyperactivation of IRF5 has been implicated in numerous autoimmune diseases, chief among them systemic lupus erythematosus (SLE). SLE is a heterogeneous autoimmune disease in which patients often share similar characteristics in terms of autoantibody production and strong genetic risk factors, yet also possess unique disease signatures. *IRF5* pathogenic alleles contribute one of the strongest risk factors for SLE disease development. Multiple models of murine lupus have shown that loss of *Irf5* is protective against disease development. In an attempt to elucidate the regulatory role(s) of IRF5 in driving SLE pathogenesis, labs have begun to examine the function of IRF5 in several immune cell types, including B cells, macrophages, and dendritic cells. A somewhat untouched area of research on IRF5 is in T cells, even though *Irf5* knockout mice were reported to have skewing of T cell subsets from T helper 1 (Th1) and T helper 17 (Th17) toward T helper 2 (Th2), indicating a potential role for IRF5 in T cell regulation. However, most studies attributed this T cell phenotype in *Irf5* knockout mice to dysregulation of antigen presenting cell function rather than an intrinsic role for IRF5 in T cells. In this review, we offer a different interpretation of the literature. The role of IRF5 in T cells, specifically its control of T cell effector polarization and the resultant T cell-mediated cytokine production, has yet to be elucidated. A strong understanding of the regulatory role(s) of this key transcription factor in T cells is necessary for us to grasp the full picture of the complex pathogenesis of autoimmune diseases like SLE.

## Introduction

T cells are responsible for balancing a variety of regulatory and effector functions. Many of these roles are accomplished through the expression of a panel of cytokines controlled by a specific cohort of transcription factors. These cytokines can act to initiate, support or inhibit different T cell effector functions and, during homeostatic conditions, maintain a tight immunological balance between pro- and anti-inflammatory T cell functions. In the case of immune-mediated diseases, the balance between T cell subsets is often disrupted. For instance, patients with systemic lupus erythematosus (SLE) demonstrate an increase in T helper 1 (Th1) relative to T helper 2 (Th2) cells and a dysregulated balance between Th1 and T helper 17 (Th17) cells, while results from single cell sequencing of patients with rheumatoid arthritis (RA) have demonstrated a skewing toward Th1 effector memory CD4+ T cells, and a murine model of multiple sclerosis (MS) showed resistance to disease development due, in part, to a loss of key T cell intrinsic Th1 mediators (1–4). In SLE studies performed in humans and mice, some of the likely cytokine inflammatory mediators and immunomodulatory agents identified as participating in disease development include (but are not limited to) interferon (IFN)-α, IFN-γ, tumor necrosis factor (TNF), interleukin (IL)-1, IL-2, IL-4, IL-6, IL-8, IL-9, IL-10, IL-12, IL-13, IL-17, IL-21 and transforming growth factor (TGF)-β (5–8). Although many of these cytokines are produced by various antigen presenting cells (APCs) to help initiate effector T cell responses, all of these cytokines are also produced in varying quantities by the effector T cells themselves. Sustained T cell response, both appropriate and pathogenic following the initial priming event, depends greatly on the ability of the T cells to both produce the appropriate cytokines and reformat their transcriptional landscape at an epigenetic level to generate a positive feedback loop. Dysregulation of this positive feedback loop or inappropriate epigenetic reprogramming could result in a T cell-driven dysregulation of pro- or anti-inflammatory cytokine production, as seen in numerous autoimmune diseases (9–11). This review will delve specifically into the potential roles for IRF5 in the regulation of effector T cell decision and maintenance with a focus on Th1, Th2 and Th17 subsets, whose high interconnectivity has been demonstrated to be impacted by IRF5 deletion or hyperactivation. However, continued research into a potential role(s) for IRF5 in the other T cell subsets, particularly follicular helper T (Tfh) cells and regulatory T (Treg) cells, is an important next step in the elucidation of autoimmune disease pathogenesis via IRF5 dysregulation. For a more general review on the role of cytokines in autoimmune disease, see Raphael et al. (12).

### Th1 Cells

Th1 effector cells regulate the body's defense against viruses, bacteria and intracellular pathogens and, when properly functional, are vital members of the immunological homeostasis required to maintain our health. However, dysregulation of Th1 cells has been implicated as a key player in the global immunological dysfunction that results in many autoimmune disease conditions, among them RA, SLE, MS, type 1 diabetes mellitus, idiopathic thrombocytopenic purpura, and experimental allergic encephalomyelitis (1, 2, 13–19). Th1 cells are traditionally defined by their production of IL-2 and IFN-γ and by expression of the transcription factor and epigenetic modifier, T-bet, a member of the T-box family of transcription factors (20–22). In the subsequent decades following the initial characterization of these defining factors, critical roles for the DNA-binding regulatory proteins signal transducer and activator of transcription 4 (STAT4), STAT1, and STAT5 in the development and support of Th1 subsets have also been revealed (23, 24). Briefly, naïve CD4+ T cells are stimulated to develop into Th1 effector cells by IL-12 binding to the IL-12 receptor (23, 25). Once activated, Th1 cells produce IL-2 and IFN-y. IL-2 acts as a potent inducer of both T cell proliferation and T cell effector fate decision (26). IFN-y employs both stimulatory and inhibitory roles to maintain Th1 effector dominance. IFN-y can induce the phosphorylation of STAT1, thereby increasing expression of the Th1 specific genes, IL-12 receptor beta 1 (*IL12RB*) and T-box transcription factor 21 (*TBX21*; encoding T-bet). Increased levels of T-bet results in positive feedback on T-bet expression through T-bet activation of *IFNG* transcription. T-bet also increases STAT1 activation and mediates the upregulation of Th1-specific genes including *IL12R*, which will in turn signal to increase STAT4 phosphorylation and dimerization. STAT4 itself can act as a potent transcriptional repressor of genes that would normally support Th2 differentiation (i.e., *GATA3*) and acts in concert with T-bet to promote the positive feedback loop resulting in increased IFN-γ production.

This feedback loop enhancing Th1 differentiation also has built in inhibitory mechanisms. T-bet can bind to and inhibit BCL-6 (B-cell lymphoma 6 protein) early in Th1 polarization, preventing transcription of genes involved in alternative effector fates (27). T-bet and BCL-6 comprise two lineage-defining factors that cooperate in the regulation of Th1 gene expression patterns (28). However, later in Th1 activation T-bet recruits BCL-6 to the *IFNG* promoter, resulting in inhibition of *IFNG* transcription and thereby shutting down one of the main drivers of the Th1 effector response (23, 28, 29). In addition, T-bet increases the transcription of the membrane protein T cell immunoglobulin mucin-3 (Tim-3) in later stages of Th1 differentiation, which acts as an inhibitor of the Th1 response upon binding to the ligand, β-galactosidase-binding lectin 9 (Gal-9) (30, 31). Gal-9 regulates Th-induced proinflammatory cytokine production (32). Further supporting the concept that dysregulation of T-bet can result in a pathologically imbalanced immune system, Tim-3 blockade has been shown to result in autoimmune disease development (33). Interestingly, most of T-bet's transcriptional regulatory capabilities have been shown to occur through epigenetic modifications of genetic loci using either H3K4 (activating) or H3K27me3 (inactivating) chromatin methylation patterns. In fact, production of the key Th1 driving cytokine IFN-γ is dependent on both chromatin remodeling by T-bet and increased IL-12R expression through direct T-bet transcriptional activity (29, 34–36). However, much less has been published with regards to the direct negative regulation of T-bet activity in activated Th1 cells and how dysregulation at the level of T-bet could result in rampant Th1 activation and the development of autoimmune disease.

As previously described, T-bet clearly plays an indispensable role in the positive feedback loop governing Th1 effector subset polarization. T-bet both positively regulates ~50% of Th1-specific genes and inhibits Th2-specific gene transduction, including GATA3, the Th2-specific transcription factor (29). Interestingly, ~70% of Th2-specific genes in Th1 cells are still bound by GATA3. In this scenario, GATA3 is bound by T-bet and inhibited from transducing Th2-specific transcripts in Th1 effector cells (37, 38). Other sources show that T-bet can also directly interact with and recruit GATA3 away from its Th2 gene loci. In either case, it is hypothesized that part of the rationale for skewing toward a Th2 phenotype upon loss of negative regulation by *TBX21* is due to both increased *GATA3* transcription and increased GATA3 association with Th2-specific genetic loci (29).

### A Conserved DEF6-IRF5-T-bet Regulatory Axis Mediates Th1 Effector Response Through T-bet

Th1 cells are capable of producing the cytokines granulocyte macrophage colony stimulating factor (GM-CSF), IL-2, TNF-β, and IFN-γ (39). As previously described, uncontrolled positive feedback of these cytokines on T cells can result in an imbalance between T cell subsets and their secreted cytokines, resulting in the development of autoimmune disease pathologies (40). Here we will explore the role of IRF5 in regulating an appropriate Th1 immune response and how loss of IRF5 may cause effector T cell dysregulation.

In the full-body *Irf5* knockout (KO) mouse, the majority of studies have shown that there is skewing of T cells toward a Th2 effector phenotype with an accompanying decrease in Th1 effector subsets, thereby implicating a role for IRF5 in Th1 effector T cell commitment and/or maintenance (41–44). However, the T cell intrinsic IRF5-dependent molecular and genetic systems at play in these regulatory mechanisms governing Th1 feedforward and inhibitory loops have yet to be thoroughly explored. Based on previously published work, it seems likely that a main target for the dysregulation of Th1 effector T cells resulting in a substantial decrease in Th1 effector fate decision and a concomitant increase in Th2 cells would involve dysregulation of the master transcriptional regulator, T-bet. However, IRF5 does not play a role in the direct transcriptional regulation of this key transcription factor (45). This does not preclude the possibility that IRF5 interacts with T-bet on a protein level. In the following paragraphs, we propose a novel DEF6-IRF5-T-bet regulatory mechanism that controls Th1 effector T cell polarization.

**Figure 1:**
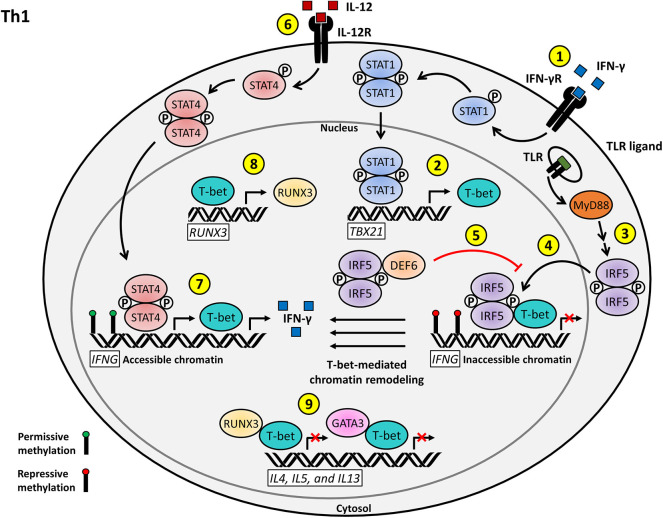
Proposed model for the T cell-intrinsic role of IRF5 as a positive regulator of Th1 effector function and differentiation. (1) IFN-γ stimulation of the IFN-γR on naïve CD4+ T cells induces STAT1 activation and nuclear translocation. (2) Phosphorylated STAT1 activates the transcription of *TBX21*, leading to the production of T-bet. (3) T cell stimulation, possibly through TLR signaling, induces IRF5 activation and nuclear translocation. (4) Nuclear IRF5 recruits T-bet to the silenced *IFNG* locus to facilitate permissive T-bet-mediated chromatin remodeling. (5) DEF6 binds to nuclear IRF5 in order to inhibit IRF5-mediated T-bet recruitment to the *IFNG* locus. (6) IL-12 signaling through the IL-12R results in STAT4 activation and nuclear translocation. (7) Phosphorylated STAT4 and T-bet induce the transcription of the accessible *IFNG* locus and subsequent IFN-γ signaling drives Th1 effector differentiation. (8) T-bet also acts as a positive regulator of *RUNX3* transcription. (9) T-bet interacts with RUNX3 and GATA3 to inhibit the transcription of Th2 signature genes, including *IL4, IL5*, and *IL13*, to promote Th1 polarization.

The SWEF family of Rho-GTPase regulatory proteins consists of two family members, switching B cell complex subunit (SWAP70) and DEF6 (also known as IRF4 binding protein, IBP, or SWAP70-like adaptor of T cells, SLAT) (46, 47). Recent publications have shown that SWAP70 and DEF6 (also recently identified as a potential risk variant in human SLE) bind to and sequester IRF5 in the nucleus of age-associated B cells (ABCs) (46, 48). In naïve CD4+ T cells, the predominant SWEF family member expressed is DEF6 (47). As in ABCs, the importance of DEF6 as a master regulator has become increasingly evident through continuing discoveries of its roles in many aspects of T cell regulation, including IRF4 modulation, cytoskeletal kinetics and protein expression control through mammalian target of rapamycin complex 1 (mTORC1) regulation (49–53). In addition, as also observed in ABCs, upon T cell stimulation IRF5 levels are shown to dramatically increase (43, 54). If a similar pattern is followed in T cells as in ABCs, elevated levels of IRF5 may allow it to escape inhibition by DEF6 and perform its crucial regulatory role(s) in T cells (Figure 1).

So what regulatory role(s) might IRF5 play in Th1 cells? Upon stimulation of ABCs with IFN-γ and IL-21, levels of IRF5 increase, thereby allowing IRF5 to escape its negative regulation by SWEF family members, translocate to ABC transcriptional sites, and recruit T-bet to ABC-specific T-bet binding motifs (46, 55). It would be interesting to examine if this similar chromatin remodeling by T-bet followed by transcriptional activation resulting specifically from IRF5 driven T-bet recruitment in ABCs is a conserved mechanism for epigenetic and transcriptional regulation in T cells. In this scenario, IRF5 deletion would also likely result in GATA3 release from T-bet, allowing increased GATA3 translocation and binding to Th2-specific cytokine promoters, resulting in increased Th2-specific genes and cytokines (Figure 1). A mechanism similar to this one has already been alluded to in the *Irf5*^−/−^ pristane-induced model of lupus, where loss of *Irf5* results in an increase in the production of the Th2-specific cytokines IL-4 and IL-5 (41, 42, 56).

The proposed inclusion of IRF5 in the regulation of T-bet through a conserved interaction with DEF6 in T cells will likely have direct implications in our understanding of the control of cytokine release by T cells and the T cell-driven pathogenesis of several autoimmune diseases. An example of this is regulation of the runt-related transcription factor 3 (*RUNX3*) gene. RUNX3 enhances IFN-γ production and inhibits IL-4 production when recruited to the promoter regions by its interaction with T-bet or other members of the T-bet family. Upon Th1 activation, RUNX3 has been shown to be transcriptionally activated by T-bet. RUNX3 will then form a complex with T-bet and translocate to the promoters of IFN-γ and IL-4, activating and inhibiting their transcription, respectively, to maintain the Th1 positive feedback loop. Our hypothesis is that T-bet recruitment to sites of transcriptional regulation is mediated by IRF5 (Figure 1). In a 2016 paper examining effects of *Runx3* polymorphisms, they identified an IRF4 binding site upstream of the *Runx3* promoter (57). In 2019, this same group identified that this area in the promoter region could also mediate binding of other transcription factors, including IRF5 (57). Loss of *Runx3* compromises IFN-γ production and abrogates inhibition of IL-4, thereby implying a vital role for RUNX3 in maintaining effector T cell polarization (58, 59).

Many key regulatory signaling and transcriptional proteins are expressed in both B and T cells. If IRF5 is indeed required to recruit T-bet to its transcriptional loci, loss of *IRF5* would result in decreased efficiency of T-bet initiation of its Th1 transcriptional program, leading to a defect in Th1 effector subset polarization, as seen in *Irf5* KO models. In addition, dysregulation of DEF6 could directly impact regulation of the key Th1 transcriptional driver, T-bet, providing a mechanism by which *DEF6* polymorphisms contribute to SLE risk (Figure 1).

### Th2 Cells

Th2 effector T cells are involved in the defense against parasitic infections, allergic reactions and the resolution of chronic inflammation (60). Unlike the previously discussed Th1 effector cells, the mechanisms driving Th2 differentiation are still not fully understood. Dendritic cells are thought to play a distinct role in supporting Th2 effector decision. However, they are incapable of producing the key Th2 mediating cytokine, IL-4 (61). Interestingly, IL-4 produced by CD4+ T cells themselves has been shown to be sufficient in initiating the Th2 response (62). These findings support the hypothesis that Th2 effector decision may be a default response in conditions where there is a lack of stimuli driving other Th effector fates. High levels of GATA3 expression in naïve CD4+ T cells prime the cells for Th2 differentiation, providing additional evidence for this theory. GATA3 is only downregulated upon initiation of T effector cell polarization into alternative subsets (61, 63).

Upon initiation of Th2 polarization, the principle Th2 cytokine, IL-4, acts in a stimulatory capacity through activation of STAT6 phosphorylation. Phosphorylated STAT6 increases the transcription of *IL4* and *GATA3*. GATA3 is both a vital component of the machinery required for *IL4, IL5*, and *IL13* transcription and is required for the global epigenetic remodeling needed to achieve Th2 polarization (64). The importance of GATA3 in Th2 effector differentiation is demonstrated by the consequences resulting from loss of *GATA3*. Even in the absence of the key Th1 cytokines, IFN-γ and IL-12, lack of *GATA3* drives Th1 polarization (65).

Increased chromatin accessibility mediated by GATA3 both leads to the secretion of the Th2 specific cytokines, IL-4, IL-5, and IL-13, and inhibits the production of the Th1 specific cytokine, IFN-γ. Interestingly, IRF4 has recently been shown to act as an additional positive regulator of *IL4* transcription during Th2 differentiation (49, 66). IRF4 forms a complex with GATA3 and the chromatin organizer special AT-rich binding protein 1 (SATB1) in order to bind to the RHS6 sequence during Th2 differentiation, located ubiquitously throughout the Th2 cytokine locus. All three of these factors are required in order for Th2-specific genes to be expressed (67). GATA3 has also been proposed to act in a positive feedback loop through the induction of IRF4 (68). The emerging roles of the complex transcriptional and regulatory networks involving the master transcription factor and epigenetic modulator, GATA3, are still being explored.

As in Th1 cells, there are regulatory mechanisms in place to inhibit the transcription of alternative T effector subset mediators upon Th2 effector commitment. One of these factors is the ubiquitous regulator, Ikaros (a hemopoietic-specific zinc finger protein also known as IKZF1). Regulatory functions for Ikaros have been implicated in almost all T helper cell subsets and loss of *Ikaros* has been shown to be detrimental in the maintenance of Th2 commitment. In *Ikaros*^*null*^ CD4+ T cells there is general hypoacetylation of the Th2 cytokine locus, increased IFN-γ production in Th2 polarizing conditions, decreased production of IL-4, IL-5, and IL-13, decreased GATA3 and c-MAF expression, and increased levels of T-bet and STAT1. All of these factors result in a skewing from Th2 to Th1 (69). Despite this growing pool of knowledge on the regulatory mechanisms ascribed to Ikaros, very little is known about the regulation of Ikaros itself in T cells (60, 70). However, a recent study of Ikaros regulation in B cells may provide insight into a conserved IRF5-dependent Ikaros regulatory mechanism in T cells (45).

### A Conserved MyD88/IRF4/IRF5 and Ikaros Regulatory Mechanism Mediates the Th1-Th2 Balance

As previously discussed, the regulation of Th2 cells, and thus the closely related Th1 effector subset, is still not fully understood. Pathologic skewing toward a Th2 response has been shown to result in atopic disorders, such as systemic sclerosis, and immunosuppression through the dysregulated production of their hallmark cytokines, IL-4, IL-5, IL-6, IL-9, IL-10, and IL-13 (40). IL-4, IL-10, and IL-4-induced IL-10 production in particular, has an inhibitory role on Th1 effector cells, thereby further contributing to the skewing from a Th1 to a Th2 phenotype and mediation of Th1 effector response (71, 72). Here we will explore a potential role for IRF5 in the control of Th2 subsets and how dysregulation can contribute to an enhanced pathogenic Th1 effector response.

A key role for IRF4 in Th2 subset development and, more specifically, the control of IL-4 production, has previously been identified (73). Although the precise regulatory mechanisms at play for IRF4 in T cells have yet to be fully elucidated, levels of IRF4 have been shown to be higher in resting Th2 cells compared to Th1 and Th17 (49). Inquiries into the role of IRF5 in other immune cell types have revealed alternative roles for IRF4 outside that of direct transcriptional regulation. In macrophages, IRF4 has been established as a negative regulator of Toll-like receptor (TLR) signaling by directly competing with IRF5 for binding to myeloid differentiation primary response gene 88 (MyD88) (74). MyD88 acts as a scaffold protein where IRF5 can receive its post-translational modifications from its interacting modulators. MyD88 functions downstream of all TLRs except TLR3 (75). Inhibition of the IRF5-MyD88 interaction by IRF4 results in attenuation of inflammatory cytokine production downstream of TLR signaling (48, 74, 76). However, the impact of T cell TLR signaling on intrinsic CD4+ T cell effector function and the pathological conditions that result from dysregulation are still by and large unconfirmed (77, 78).

**Figure 2:**
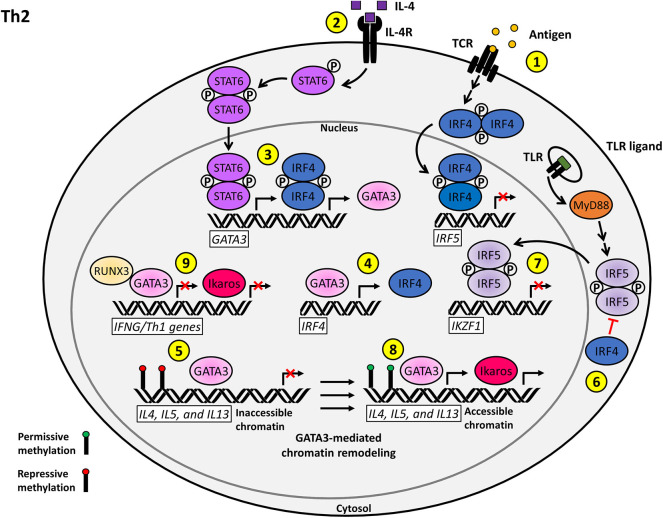
Proposed model for the T cell-intrinsic role of IRF5 as a negative regulator of Th2 effector function and differentiation. (1) Antigenic stimulation of the TCR induces the upregulation of IRF4, which acts as a repressor of *IRF5* transcription. (2) Stimulation of the IL-4R by IL-4 on naïve CD4+ T cells induces STAT6 activation and nuclear translocation. (3) Phosphorylated STAT6 synergizes with IRF4 to activate *GATA3* transcription. (4) A positive regulatory loop supported by GATA3-mediated *IRF4* transcription augments Th2 polarization. (5) GATA3 induces permissive chromatin remodeling at the *IL4, IL5*, and *lL13* Th2 cytokine locus. (6) IRF4 inhibits the TLR-induced activation of IRF5 via antagonizing the interaction between MyD88 and IRF5. (7) Nuclear IRF5 functions as a negative regulator of *IKZF1* transcription, which limits the production of Ikaros. (8) Ikaros and GATA3 promote the transcription of accessible Th2 polarizing genes including *IL4, IL5*, and *IL13*. (9) Ikaros and GATA3 further reinforce the Th2 phenotype via repression of the Th1 transcriptional network.

The main body of research on the impacts that dysfunction of TLR signaling at the level of MyD88 might have on T cells examined how loss of *MyD88* in upstream signaling cells (macrophages and dendritic cells) impacted Th2 differentiation. Little has been done to examine the specific roles and pathways of TLR signaling in T cells (75, 79). Mounting evidence implicates the TLR/MyD88 pathway as a potential regulatory mechanism in the Th1/Th2 effector decision. A study performed using the *B. burgdorferi* model of infection in a T cell-specific *MyD88* deletion model demonstrated that loss of *MyD88* in T cells results in an intrinsic defect in the Th1 and Th17 response. Th2 effector response was unfortunately not examined (80). However, an OVA-based murine *MyD88*^−/^- model of asthma showed significant defects in Th2 effector response upon stimulation (81). Taking these findings into account, we postulate that MyD88 plays an intrinsic role in T effector cell differentiation alongside IRF4. While IRF4 is expressed at high levels in Th2 effector cells, low levels of IRF5 are associated with a Th2 response. In Epstein-Barr Virus (EBV)-infected B cells, IRF4 was shown to be a negative transcriptional regulator of *IRF5* (82). If *IRF5* is no longer transcribed at high enough levels to initiate a transcriptional response tailoring an alternative T cell fate through TLR signaling, a Th2 transcriptional profile maintained by IRF4 through the previously described mechanisms can be maintained. This theory is supported by the T cell-specific *MyD88* KO. Here, removal of another key player in the IRF5 TLR signaling pathway results in Th2 skewing, akin to the results seen in the *Irf5* KO (80). Along with the conserved expression of IRF4 and IRF5 and the as of yet undefined mechanism by which IL-4 is initially regulated, we postulate that a conserved IRF4/IRF5/MyD88 axis in effector T cells may be playing a role in IRF5 activation and the skewing between Th1 and Th2 subsets (Figure 2).

Ikaros is another mediator that plays an important role in the maintenance of Th2 effector subset decision. Ikaros is a hematopoietic transcription factor that directly associates with Th2 regulatory gene loci and is involved in the positive regulation of Th2 gene expression (69). Ikaros has a binding site in its promoter region for IRF4, IRF5, and IRF8. In B cells, IRF8 and IRF5 both bind and regulate the *Ikzf1* promoter, IRF8 acts in an activating capacity while IRF5 acts as an inhibitor. Inhibition of *Ikzf1* transcription by IRF5 allows for the assumption of B cell antibody class switching to IgG2a/2c (45). Expression and function of these IRF transcription factors and Ikaros are conserved in T cells. If IRF5 were to maintain a similar negative regulatory function for Ikaros as seen in B cells, loss of *IRF5* would allow unimpeded Ikaros activation, resulting in a shift toward Th2 effector polarization. On the other hand, overexpression or hyperactivation of IRF5, as seen in SLE patients, could lead to loss of Ikaros transcription and a shift from the Th2 to Th1 effector T cell subset. In support of this theory, Gene Ontology shows that *IKZF1* has distal sites for T-bet and GATA3 binding (37). As previously discussed, we postulate that there is likely a role for IRF5 in the regulation of T-bet through direct interaction, as well as one for GATA3, by extension (Figure 2). Thus, there is increasing circumstantial evidence of a regulatory role for IRF5 in the control of Ikaros function, either through direct binding or through the recruitment of chromatin remodeling agents.

### Th17 Cells

T cell development is highly dependent on the surrounding cytokine environment and is characterized by high degrees of plasticity which, in many cases, can serve a pathogenic role. This is especially seen in the case of dysregulated Th17 cells, which have been associated with many immunological diseases including RA, inflammatory bowel disease (IBD), SLE, MS, psoriasis and cancer (20, 83, 84). Although Th17 effector subsets have been considered for drug targets to counteract the dysfunctional immune systems that they help to support, our lack of knowledge about the pathways regulating the polarization of these cells toward pathogenic phenotypes has hindered our choice(s) of a specific target (83). Recently, the monoclonal antibody against IL-17R, marketed under the name Brodalumab (AMG827), has entered clinical trials and was shown to be effective in improving psoriasis (85). However, many other drugs on the market attempting to initiate an IL-17 blockade have been met with mixed results depending on the disease setting (86). Thus, although Th17 effector function is strongly implicated as a potential target for future drug development, we need to gain a better understanding of the mechanisms controlling Th17 pathologic phenotypes and how these can drive autoimmune disease.

In SLE patients, it has been shown that hyperactive IRF5 results in skewing toward a Th1 and Th17 phenotype. However, to say simply “Th17 phenotype” is an oversimplification of the diversity of this particular T cell subset. Th17 effector T cells exist in a gradient between classical and pathogenic which is determined in part by the cytokine milieu they are exposed to. In the pathogenic state, there are two opposing directions that Th17 cells can follow—either toward a Th1-like phenotype, which is often associated with autoimmunity or toward a more Th2-like state, which is correlated with enhanced immunosuppression (83). At steady state, Th17 cells differentiate into Tfh cells and support immunoglobulin A (IgA) production by germinal center B cells. IL-23 in particular, although not required for Th17 differentiation, is required for pathogenic Th17 maintenance and survival (87).

IL-17A, the “pathogenic” cytokine produced by Th17 cells, has been shown to be a key player in the perpetuation of inflammation associated with autoimmune tissue damage. IL-17A functions through several mechanisms including the activation of other immune cells, increasing B cell functions, recruiting neutrophils, Treg mediation and enhancing proinflammatory cytokine release (20, 88, 89). In the mouse model of human MS (murine experimental autoimmune encephalomyelitis, EAE), blocking the interaction between IL-17 and IL-17 receptors resulted in substantial attenuation of EAE development (90). Unfortunately, the picture painted by this interaction is oversimplified. To date, there have been six different IL-17 cytokines identified, IL-17A–F, and five unique versions of the IL-17 receptor, IL-17RA–RE. For a more extensive review on what is known about the functional variations of these family members, see Swaidani et al. (91) and Jin and Dong (92). Although IL-17A has been identified as the main mediator of inflammation associated with autoimmune disease, the pathways downstream of IL-17A binding to IL-17R are still not fully defined.

In non-disease states, Th17 cells serve an important function in supporting tailored immune responses to various pathogens (20). Th17 effector cells maintain a balance between the alternative Treg differentiation pathway and conversion into a Th1-like phenotype. IRF4 has been shown to be a key mediating factor in maintaining the balance between Th17 and Tregs. *Irf4* KO results in an increase in the Treg FoxP3 (forkhead box P3) transcription factor and a decrease in RORγt (RAR-related orphan receptor gamma t), the major transcription factor for commitment to Th17 fate in part through transcriptional upregulation of IL-17 (93). The relatively one-sided conversion from Th17 to Th1 seems to be controlled through stimulation from circulating cytokines. Stimulation of Th17 polarized cells by IL-12 and IFN-γ results in inhibition of IL-17 secretion and conversion to a more Th1-like state, characterized by increased levels of STAT4 and T-bet expression. Increased levels of TGF-β inhibit this plasticity and result in maintenance of a stable Th17 phenotype. Early STAT transcription factors are also at play in the regulation of Th17 decision; STAT3 promotes and STAT5 inhibits Th17 differentiation (23). Because of the plasticity of the Th17 subset and its ability to interconvert between many other effector-like subsets in response to disease, the regulation of this particular subset is complex and still not well-understood. However, it has been established that maintenance of the inflammatory state that characterizes many autoimmune diseases is in part due to the IL-17-initiated positive feedback loop from defective Th17 cells (94, 95).

### A Potential IRF5-Mediated T Cell-Intrinsic Feedback Loop Regulates the Th17 Effector Decision Through Inflammatory Cytokine Production, STAT3, Ikaros and IL-10

The role of IRF5 in Th17 effector cells is still an open field. However, based on the previous mechanisms described, especially those relating to Th1 regulation, a role for IRF5 in Th17 differentiation and plasticity seems highly likely. Several studies have supported a role for IRF5 in Th17 effector differentiation, although few, if any, studies have yet to examine an intrinsic role for IRF5 in Th17 cells. Loss of *Irf5* in murine models of severe asthma resulted in decreased IFN-γ and IL-17 responses upon ovalbumin (OVA) immunization (96). In an *Irf5* KO antigen-induced arthritis (AIA) model, Th1, Th17, and γδ IL-17 producing T cells were found to have significantly decreased effector responses following immunization with methylated bovine serum albumin (mBSA) in complete Freund's adjuvant (CFA). In addition, this model showed decreased levels of *Ifng* and *Il17a* mRNA and the key Th1 and Th17 cytokines IL-1β, IL-6, IL-12, and IL-23 (97).

The cytokines that are often used to characterize pro-inflammatory Th17 subsets are IL-22, GM-CSF, and IFN-γ. Interestingly, several of these inflammatory cytokine mediators are also known to induce the expression of IRF5. In macrophages, increased IRF5 expression results in an M1 (inflammatory) macrophage phenotype through the upregulation of IL-12, TNF-α, and IFN-γ, with concomitant repression of IL-10 (98). Through binding to various promoter regions, IRF5 also increases *IL6, IL12*, and *IL23p19* transcription. Interestingly, pathogenic Th17 cells also secrete IL-12, IL-23, IL-6, and IFN-γ in addition to various other Th17-specific effector cytokines and transcription factors (66, 99). A conserved role for IRF5 in the transcriptional activation of these inflammatory cytokines in Th17 cells should be explored.

**Figure 3:**
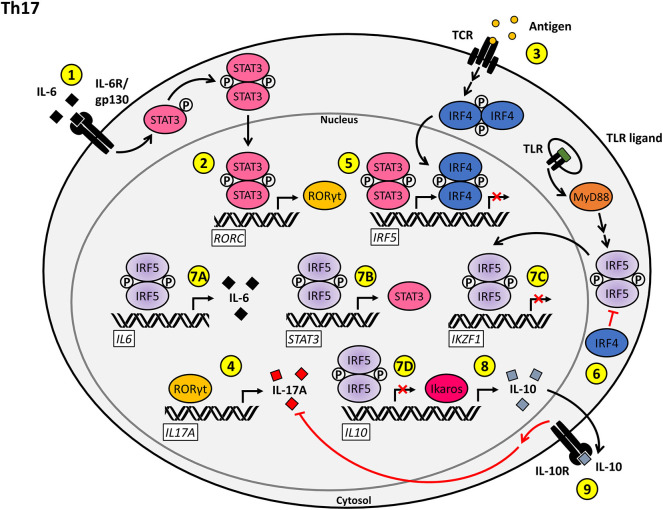
Proposed model for the T cell-intrinsic role of IRF5 as a positive regulator of Th17 effector function and differentiation. (1) Stimulation of the IL-6R/gp130 complex by IL-6 on naïve CD4+ T cells induces STAT3 activation and nuclear translocation. (2) Phosphorylated STAT3 induces the transcription of *RORC*, leading to the production of RORγt. (3) Antigenic stimulation of the TCR induces the upregulation of IRF4. (4) RORγt induces the transcription of *IL17A*, driving Th17-mediated inflammation. (5) The transcription of *IRF5* is positively regulated by phosphorylated STAT3 and negatively regulated by IRF4. (6) IRF4 inhibits the TLR-induced activation of IRF5 via antagonizing the interaction between MyD88 and IRF5. (7A–D) Nuclear IRF5 promotes the Th17 phenotype through upregulation of *IL6* (7A) and *STAT3* (7B) expression and repression of *IKZF1* (7C) and *IL10* (7D) transcription. (8) Ikaros functions as a positive regulator of *IL10* transcription. (9) IL-10 signaling through the IL-10R antagonizes the production of IL-17A, thereby inhibiting Th17 inflammatory responses.

IL-10 production by Th17 cells may provide yet another avenue for a potential role for IRF5 in the regulation of Th17-mediated inflammation (100). Although the regulation of IL-10 in Th17 effector cells is not fully understood, it has been well-established that IL-10 is required for T cells to maintain control over Th17 effector function (101). One of the mechanisms by which IL-10 expression is mediated is through TGF-β and IL-6. These two factors work to activate the c-MAF transcription factor through STAT3, which in turn activates *IL10* transcription by binding to the *IL10* promoter (102). IL-10 acts to reduce IL-17 and IFN-γ production, thus negatively regulating pro-inflammatory Th17 effector reactions. Interestingly, in the context of Newcastle disease virus (NDV)-infected Balb-C mice, IRF5 was shown to induce *Stat3* transcription in the presence of undetectable levels of the cytokines IL-6 and IL-10 (103). In addition, IRF5 has been shown to be a key mediator of *IL6* transcription in human pDCs (104). IRF5 was also shown to be upregulated by the janus kinase 2 (JAK2)/STAT3 pathway in human umbilical vein endothelial cells (105). The existence of a positive feedback loop between STAT3 and IRF5 in Th17 cells, where activation of IRF5 transcription downstream of STAT3 allows for IRF5 to feedback and increase IL-6 and STAT3 expression, should be explored as a potential mechanism by which IRF5 mediates Th17 effector response (Figure 3).

In an alternative regulatory arm, Ikaros has been shown to be required for inhibition of heterochromatic remodeling at the gene loci for the Th17 effector program. Ikaros has also been shown to repress expression of both *FOXP3* and *TBX21*, which both normally act to negatively regulate Th17 development (106, 107). However, T-bet was also reported to positively regulate transcription of the *IL23R* by binding to a specific site in the *IL23R* promoter sequence and inducing IFN-γ expression by Th17 cells, thus inducing a pro-inflammatory state (108, 109). Hence, depending on the location and context of T-bet expression in Th17 cells, T-bet can initiate or ameliorate inflammatory responses. The precise mechanisms through which these regulatory actions are achieved have not yet been established. In addition to binding to and regulating *FOXP3* and *TBX21* expression, Ikaros has a binding site specifically within the *IL10* promoter and acts to positively regulate IL-10 production. It is likely that Ikaros has other, as of yet, undefined epigenetic and transcriptional regulatory roles to support Th17 effector functions (106, 110). A hint as to additional regulatory mechanisms involved in the pathways leading to Th17 effector commitment comes through literature on IRF5 regulation. In macrophages, IRF5 has been shown to have both positive and negative effects on *IL10* transcription through direct binding to the *IL10* promoter (98, 111). In Th17 cells, IL-10 plays a crucial role in the downregulation of the pro-inflammatory cytokines, IL-17 and IFN-γ. As loss of *IRF5* results in a decrease in Th17 effector subsets, this could imply a positive regulatory role for IRF5 in a conserved pathway, either through an inhibitory role at the *IL10* promoter (as seen in macrophages), a negative role in Ikaros regulation (as described in B cells) or induction of a STAT3–IRF5 positive feedback loop as previously described (Figure 3).

## Conclusion

The role and relevance of IRF5 in immune cell dysfunction in the context of autoimmune disease and cancer progression has become a hot topic for research in recent years. However, despite our growing knowledge of functions for IRF5 in APCs, our knowledge on the role of T cell-intrinsic IRF5 function is still lacking. Most of the literature published on potential roles for IRF5 in T cells is confounded by the dysregulation of other upstream immune cell signaling pathways in the *in vivo* setting of an *Irf5*^−/−^ mouse. The CD4+ T cell-specific *Irf5* KO model attempted to address this and, in the context of CD3/CD28 TCR stimulation with IL-12, showed no defects in IFN-γ production (54). However, preliminary work from our lab utilizing *RAG2*^−/−^ mice as recipients of *Irf5*^+/+^ and *Irf5*^−/−^ T cells reveals a stimulus-dependent T cell-intrinsic defect that drives aberrant immune cell responses which, in the context of the hypothesized TLR driven IRF5 pathways in T cells, rather than rejecting previous work, compliments their findings (data not shown). The generation and characterization of new T cell-specific conditional *Irf5* KO mice, combined with pathway-specific immune challenges, will help to delineate Irf5 intrinsic function in T cells. For example, to study an intrinsic role for Irf5 in Th17 cells, *Irf5*-floxed mice would be crossed to IL17(A/F)-cre mice to generate Th17-specific *Irf5* conditional KO mice. A number of T cell-specific cre-reporter strains are currently available that would help prove or disprove the presented hypotheses.

In the clinical realm, SLE is characterized by a heterogeneous patient population. In each patient, the disease shares several common characteristics, but ultimately has a unique landscape and response to treatment. This is likely driven by a “multi-hit” scenario where dysfunction or dysregulation of a single (or multiple) master regulatory factor, like IRF5, will predispose individuals to developing a specific brand of immune dysregulation with many shared pathological characteristics (112). However, ultimately the path of development and resulting severity of the disease is determined by the addition of other risk allelic variations, thereby leading to the unique signature characterizing individual autoimmune conditions. This also explains the as-of-yet undefined and heterogeneous pathway-specific triggers that lead to disease development in a perfect storm of self-perpetuating dysregulated pathway activation, characterized by aberrant cytokine production. Ultimately, the goal in effective therapeutic development is to find the most specific target that ameliorates the greatest number of disease phenotypes with the fewest off-target effects. In order to accomplish this, we need a detailed understanding of the pathways that govern each immune cell implicated in disease pathogenesis. Targeting the inflammatory cytokines themselves is a difficult and non-specific therapeutic option, although early clinical trials of low dose IL-2 administration have shown some promise in patients with treatment-resistant SLE (113). However, the list of “T cell” therapeutics for autoimmune disease is brief, and many of them [i.e., secukinumab, ixekizumab, broalumab, ustekinumab, iberdomide, AMG 570 targeting ICOS-L (NCT04058028)] have either yet to be proven efficacious in the treatment of SLE, are still in early clinical trials, or broadly target the functions of other immune cells (114–116). As a result, targeted delivery of therapeutic molecules to specific immune cell subsets that drive the dysregulated release of either pro-or anti-inflammatory cytokines is the future of effective personalized treatments for autoimmune disease.

## Author Contributions

ZB, MR, and BB conceived of and wrote the manuscript.

## Conflict of Interest

The authors declare that the research was conducted in the absence of any commercial or financial relationships that could be construed as a potential conflict of interest.
